# The Role of Foveal Cortex in Discriminating Peripheral Stimuli: The Sketchpad Hypothesis

**DOI:** 10.3390/neurosci4010002

**Published:** 2022-12-23

**Authors:** Carolina Maria Oletto, Giulio Contemori, Marco Bertamini, Luca Battaglini

**Affiliations:** Department of General Psychology, University of Padova, 35131 Padova, Italy

**Keywords:** foveal feedback, visual sketchpad, V1, peripheral vision, central vision

## Abstract

Foveal (central) and peripheral vision are strongly interconnected to provide an integrated experience of the world around us. Recently, it has been suggested that there is a feedback mechanism that links foveal and peripheral vision. This peripheral-to-foveal feedback differs from other feedback mechanisms in that during visual processing a novel representation of a stimulus is formed in a different cortical region than that of the feedforward representation. The functional role of foveal feedback is not yet completely understood, but some evidence from neuroimaging studies suggests a link with peripheral shape processing. Behavioural and transcranial magnetic stimulation studies show impairment in peripheral shape discrimination when the foveal retinotopic cortex is disrupted post stimulus presentation. This review aims to link these findings to the visual sketchpad hypothesis. According to this hypothesis, foveal retinotopic cortex stores task-relevant information to aid identification of peripherally presented objects. We discuss how the characteristics of foveal feedback support this hypothesis and rule out other possible explanations. We also discuss the possibility that the foveal feedback may be independent of the sensory modality of the stimulation.

## 1. Introduction

Foveal (central) and peripheral vision differ in many ways. The photoreceptor distribution is one of the most evident, with rods being absent in the central portion of the visual field while cones are scant in the peripheral part. Convergence from photoreceptors and ganglion cells also increases as eccentricity increases. Therefore, peripheral vision has lower resolution and higher crowding when compared to the central vision [1]. Yet, we do not have the impression of a central portion with blurred surrounds. Instead, we perceive the visual environment as uniform and integrated. Our brain constantly integrates the visual information coming from different sources and across different time scales thus providing a unitary percept of the visual world around us. In a recent review, Stewart et al. [2] described the interconnection between foveal and peripheral vision. It emerged that considering peripheral vision as a less precise version of foveal vision is too simplistic, and that the two are profoundly interrelated. Williams et al.’s finding shed light on one of the mechanisms that connects foveal and peripheral vision in peripheral objects discrimination: foveal retinotopic cortex is recruited in peripheral objects discrimination tasks through feedback from higher visual areas [3]. We will refer to this type of feedback as foveal feedback.

The literature on foveal feedback is still limited, yet on the basis of existing evidence we suggest that foveal retinotopic cortex acts as a sort of visual sketchpad for processing fine-detail information [3,4].

## 2. Feedback in the Classical Retinotopic Framework

Visual object recognition cannot be based exclusively on feedforward processing. There are many downward projections from higher- to lower-level areas in the visual system [5]. There is growing evidence of feedback playing an important role in visual perception [6,7].

According to some models, feedback is described as a modulation or anticipation of the feedforward generated representations of the objects [8] by carrying information about the context [9]. In predictive coding models, feedback from higher cortical areas reduces activity in the primary visual cortex (V1) when stimuli form a coherent shape. Since global shape of the stimuli is represented in higher visual areas, there is no need for lower visual areas to further process stimuli information. As a result, this context information is fed back to V1, and its activity is reduced [10,11].

In figure-ground segregation, both suppressive and enhancing feedback mechanisms have been found. Klin et al. [12] found a suppression in V1 for the figure, and less suppression for the background through feedback mechanisms. Other studies found the opposite effect [13,14]: information about the boundaries of the figure is fed forward to higher visual areas, which in turn send excitatory inputs to V1 through feedback connections [15].

A review by Cumming and Nienborg [16] suggests the existence of an anticipatory feedback employed for probabilistic inference: feedback information on prior knowledge of a task is integrated with feedforward visual input, thus influencing the responses in the task.

The literature suggests that feedback of visual information is retinotopically organized [17]: feedback and feedforward information are thought to converge on the same retinotopic area, which corresponds to the location of the object.

One exception to the retinotopically organised feedback is predictive remapping. Based on the predictive remapping hypothesis [18], the feedback may involve different retinal locations. In particular, before a saccade, neurons are active in response to information from outside their receptive field, that is, they update their response according to the future retinal location of the stimulus [19].

Williams et al.’s [3] work, on the other hand, demonstrated the existence of a special case of feedback. In foveal feedback, novel representations are formed in a different cortical region than that of the feedforward representation, but are not necessarily linked to saccade planning. The difference between predictive remapping and foveal feedback is discussed in Section 9.

## 3. Data from fMRI

**Figure 1:**
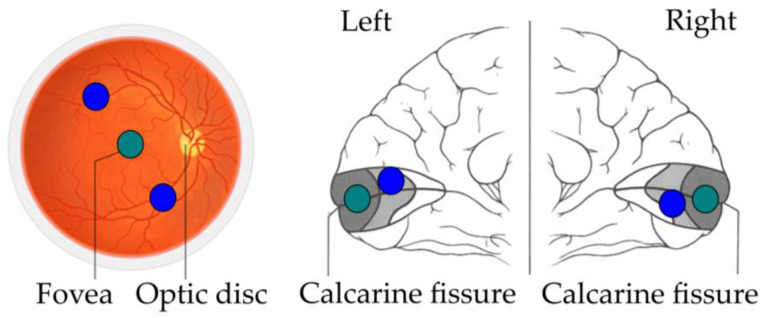
Peripheral information from the retina is forwarded to peripheral retinotopic cortex and foveal information is forwarded to foveal retinotopic cortex. Task relevant information is then fed back to foveal retinotopic cortex from higher cortical areas.

Williams et al. [3] conducted a functional magnetic resonance imaging (fMRI) study in which they analysed the foveal retinotopic activity in response to peripherally presented objects. Participants saw novel objects taken from three categories (smoothies, spikes, cubies, [20]). Two objects from the same category were displayed in diagonally opposite peripheral locations while participants were looking at the centre and performing a same/different task. The goal of this experiment was to find which area contained information for between-category discrimination using multivariate pattern analysis [21]. Surprisingly, information was found in the foveal retinotopic cortex, even if objects were presented more than five degrees from the centre of the visual field. The authors explained these findings with the existence of a feedback mechanism different from that of the classical theories: a mechanism that feeds object information from high-level object areas back to foveal retinotopic cortex to enhance task performance (Figure 1).

Further evidence of this foveal retinotopic activity for peripherally presented stimuli comes from a study by Woolgar et al. [22]. They used the same set of stimuli in a peripheral identification task in which two objects were displayed in peripherally opposing locations along the horizontal meridian. On each block, participants were told to attend one object or the other using colour cues and to judge the category of the target object. As in Williams et al.’s [3] study, foveal retinotopic activation was found, even if both the target and the nontarget object were presented in a different retinal location.

## 4. Data from TMS

Chambers et al. [23] provided causal evidence of the role of foveal feedback in performance enhancement for peripheral discrimination tasks. They used the same set of stimuli and a similar same/different task as Williams et al. [3] with a transcranial magnetic stimulation (TMS). They used a figure-of-eight coil with the handle oriented upright so that the current flow was induced along the superior–inferior axis. A double TMS pulse with 50 ms of pulse width was applied to the posterior termination of the calcarine sulcus (corresponding to the foveal retinotopic site) or to a control nonfoveal site (15 mm above the foveal site). Two sessions of TMS were performed, one for each site, with a delay of at least 24 h between them. For each session, there were eight blocks of 112 trials, in which the double TMS pulse was applied at different timings after stimuli onset (stimulus onset asynchrony SOA) and with a high vs. low intensity (120% vs. 40% motor threshold) within each block.

A drop in performance was found at 350/400 ms SOA for the high-intensity trials when the targets were presented in the periphery.

The authors argued that the effect of the disruption could be attentive. The double TMS pulse could act as spatial priming, drawing attention away from peripheral stimuli. To rule out this alternative explanation, Chambers et al. [23] applied a double TMS pulse at an SOA of 150/100 ms prior to stimuli onset. According to the authors, at this timing the priming effect should be maximal. They did not find any effect of the double pulse at 150/100 ms SOA. They concluded that the impairment found with the double pulse at 350/400 ms SOA was not due to spatial priming.

According to Chambers et al. [23], the timing-specific effect of the disruption cannot be explained by the same mechanisms that support attention [24]. These mechanisms rely on feedforward processing, while the timing at which the TMS double pulse was effective was around 100 ms later than expected for attentional modulation [25].

## 5. Behavioural Data

Some authors [4,26,27,28] tried to replicate the foveal disruption using the same peripheral discrimination task and the same stimuli as Williams et al. [3] and Chambers et al. [23]. They used a visual distractor displayed at fovea (foveal mask) in place of the TMS double pulse. The visual distractor was either a dynamic coloured square [27,28] or an object similar to the peripheral targets [4,26].

The rationale is that feedforward information from the foveal mask would interfere with the feedback information from higher cortical areas in the foveal retinotopic cortex, thus disrupting performance. That is, when feedback from peripheral stimuli and foveal mask information reach the foveal retinotopic cortex at the same time, they interfere with one another. As a result, the foveal cortex is no longer able to accurately process peripheral shape information. TMS and mask produce a similar disruptive effect on performance. Nonetheless, the way in which the disruption is achieved is very different. This is reflected in the different timings at which double TMS pulse and foveal mask have an effect on discriminating peripheral stimuli. TMS cause an immediate tetanic activation on the stimulated areas. Visual stimulus displayed at fixation takes around 40–120 ms to reach the foveal retinotopic cortex and the activation builds over time for the duration of the stimulus [29,30]. Peripheral stimuli need to reach higher visual areas before being fed back to the foveal retinotopic cortex. Therefore, the foveal mask should be displayed after the onset of peripheral targets. It is expected that a foveal mask will have a disruption effect on peripheral discrimination when presented at earlier SOAs with respect to 350/400 ms SOA found in Chambers et al.’s [23] paradigm. However, the specific time in which the disruption is effective was unclear. For this reason, in the studies mentioned below, different SOAs for foveal mask presentation were used. Table 1 summarises the characteristics of each experiment. In each study, one or two targets were displayed in the periphery. At specific SOAs, a foveal mask was displayed. Participants were then asked to perform discrimination tasks.

Weldon et al. [4] selected five levels of SOA: two (−267 and −117 ms) in which the central mask was displayed before targets onset; two (+117 and +267 ms) in which it was displayed after targets onset; one in which targets and mask were presented at the same time (0 ms). They found that the only SOA at which discriminability was impaired was +117 ms.

Fan et al. [27] presented the foveal mask at SOAs of 50, 150, 250, 350, and 450 ms after stimuli onset. Compared to a no-mask condition, performance in peripheral discrimination decreased at the 250 ms SOA. Using the same paradigm as Fan et al. [27], Contemori et al. [28] used a finer sampling of 61 levels of SOAs (from 0 to 500 ms), and estimated the dip at 94 ms.

Yu and Shim [32] introduced a slightly different paradigm. Participants were presented with a single peripheral stimulus and were asked to judge if it was a previous selected target or a different object from the same category. One of three different foveal masks was presented with a 0 and 150 ms SOA: the target, an object from another category, or a scrambled object. In contrast to the disrupting effect found in the other studies, they found an improvement in performance at the 150 ms SOA, but only when the foveal mask was identical to the target.

The timing-specific effect of the mask rules out the attentive hypothesis: if the foveal mask had an attention driving effect, it would have affected negative or early positive SOA rather than late positive SOAs. If the effect was to alert the system, it would have been present at every positive SOA. 

Ramezani et al. [31] addressed how manipulation of the foveal feedback information can affect processing of features that involve higher-level visual features and areas, such as categorization. Participants had to discriminate different category levels: superordinate level (animal vs. nonanimal, vehicle vs. nonvehicle), basic level (bird vs. nonbird, reptile vs. nonreptile, car vs. noncar, and airplane vs. nonairplane), and subordinate level (two bird tasks, two reptile tasks, two car tasks, and two airplane tasks). A central mask was presented at 100, 200, 300, and 400 ms SOAs. They found that the central mask impairs peripheral category discrimination at 300 ms SOA in the basic and subordinate levels, but not in the superordinate one.

## 6. Characteristics of the Foveal Feedback

What kind of information is fed back to the foveal retinotopic cortex is debated. Williams et al. [3] failed to find object information in the foveal retinotopic cortex in a colour discrimination task. On the other hand, Weldon et al. [26] found an impairment in a same/different colour discrimination task when presenting a foveal mask. The authors’ explanation on this discrepancy was that Williams et al. did not check for colour information in foveal retinotopic cortex; they probed shape information in both shape and colour discrimination tasks.

When using low-level feature [32] or blurred stimuli [27], there was no modulation in performance by the foveal mask. Thus, only high-level detailed object information seems to be fed back. This hypothesis is supported by fMRI data: Fan et al. [27] collected fRMI data while participants performed the peripheral discrimination task. They found that foveal retinotopic cortex contains both information about the object category and the object orientation. Thus, the representation in the foveal retinotopic cortex is more image-like, in that it contains both the general shape information (orientation in this case) and the spatial details (in this case, the features to discriminate amongst categories). It is to note that orientation could be read out only for objects in the same category. Moreover, there was no correlation between orientation and object category information. The finding that the foveal mask impairs category discrimination only for basic and subordinate categories [31] further supports this hypothesis.

The information that is fed back to the foveal retinotopic cortex is position invariant. The pattern of activation in the foveal retinotopic cortex found in the fMRI experiment by Williams et al. [3] was the same across different peripheral objects locations. Additionally, the eccentricity at which peripheral stimuli were presented varied between the studies that found an effect of the foveal mask presentation (Table 1).

In the above tasks, the eccentricity at which peripheral objects were displayed was irrelevant for the task. For this reason, information about peripheral stimuli eccentricity was not represented in foveal retinotopic cortex. Further, in a category discrimination task with natural-not-novel objects [31], the impairment was eccentricity-dependent; in the basic level (e.g., bird vs. nonbird) it was only present at 24° of eccentricity, while in the subordinate level (e.g., discriminate between two different birds) it was present at 18° of eccentricity.

Furthermore, foveal feedback is position-specific: when both peripheral stimuli were displayed in the upper or lower visual field, there was no object information in the spatial midpoint between them. The information about presented stimuli was still found in foveal retinotopic cortex [3]. When a foveal mask was added it impaired performance, even when not displayed in the midpoint of the two peripheral targets [4,27]. In addition, only a foveal mask affected performance: when the peripheral discrimination task was performed with a peripheral mask, it impaired performance at all SOAs in the same way [4,27]. When the mask was presented in a parafoveal location, in a peripheral identification task, no modulation of performance was found [32]. Owing to different representational size across fovea and periphery, a foveal mask may cause more important impairment than a peripheral one of the same size [33]. When masks were scaled for cortical magnification, the impairment in performance still occurred only for the central mask [4]. Modifying its size, the foveal mask still had a disruptive effect on performance at specific timings [27]. The specificity of the foveal retinotopic cortex disruption for the impairment in peripheral discrimination implies that this area stores task-relevant object information.

## 7. Effects of the Similarity between the Foveal Mask and Peripheral Targets

In foveal visual search, the similarity of a visual distractor with the targets are known to increase performance when targets and distractors are contemporary [33]. Weldon et al. [4] tested if this held true for peripherally presented targets by using both categorically consistent and inconsistent distractors in their experiments. They found no difference in the performance impairment between a mask from the same or a different category from the targets. On the other hand, Yu and Shim [32] found an improvement when the mask and the target were identical compared to when the mask was an object from a different category or a scrambled object. They concluded that similarity between target and mask enhances performance. However, Weldon et al. [26] still found an impairment in performance using a foveal mask of the same category as the peripheral stimuli.

These apparently opposite findings could be explained in part considering the difference in the procedures. Weldon et al. [4,26] presented two peripherally opposing stimuli and a foveal distractor, while Yu and Shim [32] only presented one peripheral stimulus and one distractor. If foveal retinotopic cortex serves as a visual storage in which the two peripheral stimuli are represented together, presenting a third stimulus will impair performance. We can see a decrease in performance whether this stimulus is of the same or different category [4], a simple coloured square [27,28,31], or whether it is grey vs. coloured [26]. The only case in which the foveal mask enhanced performance was when it was identical to the target [32]. In this case, two identical stimuli aid target recognition, because foveal retinotopic cortex receives the same information twice.

When using low-level feature objects (coloured patches, [26]), results were different for a coloured vs. a grey distractor. For the coloured mask the significant drop in performance was found at +117 ms compared to the 0 ms condition. For the grey mask the significant difference was only found at 0 ms compared to −267 ms. A possible explanation is related to the timing of the foveal distractor being flexible and dependent on a wide range of factors: duration of the target [4,26], task demanding [4,27] and number of peripheral stimuli [27]. A peripheral discrimination task with coloured patches could be too easy for a very different foveal distractor to disrupt performance. A grey scale distractor could be easily ignored while comparing peripheral colours. On the other hand, a coloured distractor is more difficult to ignore, even in a simple task. A coloured patch would act as a proper distractor in that retinotopic cortex receives information from three different colours, one irrelevant and two relevant for the task. When the patch is grey, there are only two colours to be compared.

## 8. Is Foveal Feedback Specific to Vision?

There are some studies using nonvisual stimuli and nonvisual modalities of perception that demonstrate the recruitment of foveal cortex for storing object information. Let us consider the case of spatial information coming from touching an object.

Something similar to the foveal feedback phenomenon was found for tactile–visual integration. Monaco et al. [34] found that haptic exploration of objects in the dark recruits foveal retinotopic cortex in addition to the retinotopic areas corresponding to the object position. That is, the foveal retinotopic cortex builds a representation of the object even in absence of visual information. Participants were asked to perform a delayed action on peripheral objects previously seen or touched in the dark. During grasping after a delay, both foveal retinotopic cortex and corresponding retinotopic area were activated, independently of the modality used to explore the object. The authors suggest that shape representation is stored in foveal retinotopic cortex and that this representation is independent of the sensory modality used to acquire it. Other evidence of the nonspecificity of the foveal feedback for sensory modality comes from Bola et al. [35]. They applied TMS in the same area as Chambers et al. [23] but used a task involving discrimination by touch. Participants were asked to read Braille letters in the tactile modality while TMS pulses were applied at different SOAs: 20–70–120 ms, 120–170–220 ms, 220–270–320 ms, 320–370–420 ms, or 420–470–520 ms. The accuracy decreased when the TMS was applied on the foveal retinotopic cortex at the 120–220 SOAs.

## 9. The Visual Sketchpad Hypothesis

The timing at which foveal disruption results in a dip in performance is not consistent with a feedforward process disruption [36]. It is unlikely that the purpose of foveal feedback is limited to a temporal storage of images. If it were the case, object information would be found in the correspondent cortical location.

A possible explanation discussed in Fan et al. [27] and Ramezani et al. [31] is that of predictive remapping. According to the predictive remapping hypothesis, cortical areas become more responsive to visual stimuli that after a saccade will end there [19]. It is possible that foveal retinotopic cortex is engaged in anticipation of a saccade toward the object [31]. If predictive remapping is a natural tendency, it will be also activated even if participants were not allowed to make a saccade towards the periphery. As a result, foveal retinotopic cortex could receive information about the shape of the stimulus due to predictive remapping even in absence of subsequent foveation of the target. Foveal disruption was not effective when participants planned a saccade away from the stimuli [27]. Further support to this hypothesis comes from a recent study by Kroell and Rolfs [37] in which they demonstrated that when the peripheral object’s characteristics are irrelevant to the task, they are still fed back to the foveal cortex: when participants are asked to report central target orientation after executing a saccade towards a peripheral probe, if target and probe have the same orientation, the accuracy is higher. Some findings are not consistent with this hypothesis. When participants had to mentally rotate the objects before performing the same/different task, the timing at which the mask affected performance shifted [27]. If information is fed back to foveal retinotopic cortex in anticipation of a saccade, there is no need for a delay in object’s foveation. Moreover, predictive remapping cannot account for the relation between performance and foveal cortical activity; in Williams et al. [3] study, performance in the peripheral discrimination task improved over blocks and was positively correlated with the activity in foveal retinotopic cortex. According to the authors, this suggests the development of a more precise mental representation of the object category, that is, foveal feedback has a behavioural relevance. It is also interesting that some studies found a shift in criterion [4,28]: following a TMS pulse or a central mask presentation, a shift towards a more conservative criterion (more “different” responses) was found. 

Another hypothesis discussed in the literature [3,4,23] is that foveal retinotopic cortex serves as a sort of visual sketchpad for task-relevant information. In the high-resolution spatial buffer hypothesis [38], V1 is used by higher-level areas when working with high-resolution detail. A link to Baddeley’s visuospatial sketchpad (VSSP) [39] can be theorized. Baddeley’s VSSP is described as storage for task-relevant visual images, which allows their manipulation in working memory. Yu and Shim [32] link foveal retinotopic cortex with the ability to retain visual features information in working memory [40] and mental imagery [41]. The VSSP can account for the relation between performance and foveal cortical activation: the more active the representation of the objects in the foveal retinotopic cortex, the better participants are at comparing them [3]. Chambers et al. [23] explained the shift in criterion by stating that the visual sketchpad aids decision making, visual working memory, and mental imagery. This is in line with a study from Kosslyn et al. [42], in which V1 is activated when participants performed a property comparison task (e.g., length of two lines) on previously seen central objects, with their eyes closed. When they applied TMS pulse to V1, performance was impaired. Other TMS findings report an impairment in a visual working memory task when TMS was applied after stimulus onset at specific timings [43,44]. Cattaneo et al. [45] tested the effect of TMS on V1 in a visual imagery task, in which participants had to imagine an analog time when presented with a digital one. TMS applied after stimulus offset disrupted performance, suggesting that V1 is involved in the encoding of the visual image. Finally, the effect of TMS disruption on visual cortex in tasks that do not imply vision [34,35] strongly supports the visual sketchpad hypothesis.

In conclusion, it is reasonable to interpret the foveal feedback phenomenon in the light of Baddeley’s visual sketchpad. Further work is needed to understand to what degree these processes overlap, and if foveal feedback is part of a broader system that encompasses visual imagery and decision making. A possibility would be to quantify the drop in performance in a paradigm similar to those described in this paper, and correlate it with the score in a visual working memory task. Foveal disruption may cause more interference for participants with a higher score in a visual working memory task.

## Figures and Tables

**Table 1:** For every study on foveal feedback with visual tasks, the following information is reported: target duration, type of foveal retinotopic cortex manipulation (TMS, mask or no manipulation for fMRI studies), timing of the dip in performance (if present and if applicable), eccentricity of the peripheral stimuli (distance from the fovea in degrees of visual angle), and the type of task performed. When nonspecified otherwise, the stimuli used in the tasks were those from [20].

Experiment	Target Duration	Manipulation	Timing of the Dip	Eccentricity	Type of Task
Chambers et al. [23]	<150 ms	TMS	350–400 ms	7	same/different
Contemori et al. [28]	100 ms	mask 83 ms	94 ms	7	same/different
Fan et al. [27]	100 ms	mask 83 ms	250 ms	7	same/different
Fan et al. blurred object [27]	100 ms	mask 83 ms	no dip	7	same/different
Fan et al. mental rotation [27]	100 ms	mask 83 ms	450–550 ms	7	same/different
Fan et al. single object [27]	100 ms	mask 83 ms	150 ms	7	same/different
Ramezani et al. categories [31]	100 ms	mask 100 ms	300 ms	0, 6, 12, 18, and 24	categorization
Weldon et al. [4]	117 ms	mask 117 ms	117 ms	6.5	same/different
Weldon et al. colour patches [26]	117 ms	mask 117 ms	117 ms	6.5	same/different
Weldon et al. coloured objects [26]	117 ms	mask 117 ms	117 ms	6.5	same/different
Williams et al. [3]	100 ms	no	-	7	same/different
Woolgar et al. category [22]	100 ms	no	-	6	categorization
Yu et al. [32]	50 ms	mask 33 ms	150 ms	10	target recognition
Yu et al. gratings [32]	50 ms	mask 33 ms	no dip	10	target recognition

## Data Availability

Not applicable.

## References

[1] Wang P., Cottrell G.W. (2017). Central and Peripheral Vision for Scene Recognition: A Neurocomputational Modeling Exploration. J. Vis..

[2] Stewart E.E.M., Valsecchi M., Schütz A.C. (2020). A Review of Interactions between Peripheral and Foveal Vision. J. Vis..

[3] Williams M.A., Baker C.I., Op de Beeck H.P., Mok Shim W., Dang S., Triantafyllou C., Kanwisher N. (2008). Feedback of Visual Object Information to Foveal Retinotopic Cortex. Nat. Neurosci..

[4] Weldon K.B., Rich A.N., Woolgar A., Williams M.A. (2016). Disruption of Foveal Space Impairs Discrimination of Peripheral Objects. Front. Psychol..

[5] Kveraga K., Ghuman A.S., Bar M. (2007). Top-down Predictions in the Cognitive Brain. Brain Cogn..

[6] Panichello M.F., Cheung O.S., Bar M. (2013). Predictive Feedback and Conscious Visual Experience. Front. Psychol..

[7] Maniglia M., Seitz A.R. (2018). Towards a Whole Brain Model of Perceptual Learning. Curr. Opin. Behav. Sci..

[8] Ress D., Heeger D.J. (2003). Neuronal Correlates of Perception in Early Visual Cortex. Nat. Neurosci..

[9] Bullier J. (2001). Integrated Model of Visual Processing. Brain Res. Rev..

[10] Murray S.O., Kersten D., Olshausen B.A., Schrater P., Woods D.L. (2002). Shape Perception Reduces Activity in Human Primary Visual Cortex. Proc. Natl. Acad. Sci. USA.

[11] Harrison L.M., Stephan K.E., Rees G., Friston K.J. (2007). Extra-Classical Receptive Field Effects Measured in Striate Cortex with FMRI. NeuroImage.

[12] Klink P.C., Dagnino B., Gariel-Mathis M.-A., Roelfsema P.R. (2017). Distinct Feedforward and Feedback Effects of Microstimulation in Visual Cortex Reveal Neural Mechanisms of Texture Segregation. Neuron.

[13] Jehee J.F.M., Roelfsema P.R., Deco G., Murre J.M.J., Lamme V.A.F. (2007). Interactions between Higher and Lower Visual Areas Improve Shape Selectivity of Higher Level Neurons—Explaining Crowding Phenomena. Brain Res..

[14] Roelfsema P.R., Lamme V.A.F., Spekreijse H., Bosch H. (2002). Figure—Ground Segregation in a Recurrent Network Architecture. J. Cogn. Neurosci..

[15] Heinen K., Jolij J., Lamme V.A.F. (2005). Figure–Ground Segregation Requires Two Distinct Periods of Activity in V1: A Transcranial Magnetic Stimulation Study. NeuroReport.

[16] Cumming B.G., Nienborg H. (2016). Feedforward and Feedback Sources of Choice Probability in Neural Population Responses. Neurobiol. Cogn. Behav..

[17] Briggs F. (2020). Role of Feedback Connections in Central Visual Processing. Annu. Rev. Vis. Sci..

[18] Duhamel J.-R., Colby C.L., Goldberg M.E. (1992). The Updating of the Representation of Visual Space in Parietal Cortex by Intended Eye Movements. Science.

[19] Melcher D. (2007). Predictive Remapping of Visual Features Precedes Saccadic Eye Movements. Nat. Neurosci..

[20] Op de Beeck H.P., Baker C.I., DiCarlo J.J., Kanwisher N.G. (2006). Discrimination Training Alters Object Representations in Human Extrastriate Cortex. J. Neurosci..

[21] Norman K.A., Polyn S.M., Detre G.J., Haxby J.V. (2006). Beyond Mind-Reading: Multi-Voxel Pattern Analysis of FMRI Data. Trends Cogn. Sci..

[22] Woolgar A., Williams M.A., Rich A.N. (2015). Attention Enhances Multi-Voxel Representation of Novel Objects in Frontal, Parietal and Visual Cortices. NeuroImage.

[23] Chambers C.D., Allen C.P.G., Maizey L., Williams M.A. (2013). Is Delayed Foveal Feedback Critical for Extra-Foveal Perception?. Cortex.

[24] Desimone R., Duncan J. (1995). Neural Mechanisms of Selective Visual Attention. Annu. Rev. Neurosci..

[25] Noesselt T., Hillyard S.A., Woldorff M.G., Schoenfeld A., Hagner T., Jäncke L., Tempelmann C., Hinrichs H., Heinze H.-J. (2002). Delayed Striate Cortical Activation during Spatial Attention. Neuron.

[26] Weldon K.B., Woolgar A., Rich A.N., Williams M.A. (2020). Late Disruption of Central Visual Field Disrupts Peripheral Perception of Form and Color. PLoS ONE.

[27] Fan X., Wang L., Shao H., Kersten D., He S. (2016). Temporally Flexible Feedback Signal to Foveal Cortex for Peripheral Object Recognition. Proc. Natl. Acad. Sci. USA.

[28] Contemori G., Oletto C.M., Cessa R., Marini E., Ronconi L., Battaglini L., Bertamini M. (2022). Investigating the Role of the Foveal Cortex in Peripheral Object Discrimination. Sci. Rep..

[29] Foxe J.J., Simpson G.V. (2002). Flow of Activation from V1 to Frontal Cortex in Humans. Exp. Brain Res..

[30] Kammer T. (2007). Masking Visual Stimuli by Transcranial Magnetic Stimulation. Psychol. Res..

[31] Ramezani F., Kheradpisheh S.R., Thorpe S.J., Ghodrati M. (2019). Object Categorization in Visual Periphery Is Modulated by Delayed Foveal Noise. J. Vis..

[32] Yu Q., Shim W.M. (2016). Modulating Foveal Representation Can Influence Visual Discrimination in the Periphery. J. Vis..

[33] Beck D.M., Lavie N. (2005). Look Here but Ignore What You See: Effects of Distractors at Fixation. J. Exp. Psychol. Hum. Percept. Perform..

[34] Monaco S., Gallivan J.P., Figley T.D., Singhal A., Culham J.C. (2017). Recruitment of Foveal Retinotopic Cortex During Haptic Exploration of Shapes and Actions in the Dark. J. Neurosci..

[35] Bola Ł., Matuszewski J., Szczepanik M., Droździel D., Sliwinska M.W., Paplińska M., Jednoróg K., Szwed M., Marchewka A. (2019). Functional Hierarchy for Tactile Processing in the Visual Cortex of Sighted Adults. NeuroImage.

[36] Lamme V.A.F., Roelfsema P.R. (2000). The Distinct Modes of Vision Offered by Feedforward and Recurrent Processing. Trends Neurosci..

[37] Kroell L.M., Rolfs M. (2022). Foveal Vision Anticipates Defining Features of Eye Movement Targets. eLife.

[38] Lee T.S., Mumford D., Romero R., Lamme V.A.F. (1998). The Role of the Primary Visual Cortex in Higher Level Vision. Vision Res..

[39] Baddeley A.D., Denis M., Engelkamp J., Richardson J.T.E. (1988). Imagery and Working Memory. Cognitive and Neuropsychological Approaches to Mental Imagery.

[40] Harrison S.A., Tong F. (2009). Decoding Reveals the Contents of Visual Working Memory in Early Visual Areas. Nature.

[41] Albers A.M., Kok P., Toni I., Dijkerman H.C., de Lange F.P. (2013). Shared Representations for Working Memory and Mental Imagery in Early Visual Cortex. Curr. Biol..

[42] Kosslyn S.M., Pascual-Leone A., Felician O., Camposano S., Keenan J.P., Ganis G., Sukel K.E., Alpert N.M. (1999). The Role of Area 17 in Visual Imagery: Convergent Evidence from PET and RTMS. Science.

[43] van de Ven V., Sack A.T. (2013). Transcranial Magnetic Stimulation of Visual Cortex in Memory: Cortical State, Interference and Reactivation of Visual Content in Memory. Behav. Brain Res..

[44] Koivisto M., Harjuniemi I., Railo H., Salminen-Vaparanta N., Revonsuo A. (2017). Transcranial Magnetic Stimulation of Early Visual Cortex Suppresses Conscious Representations in a Dichotomous Manner without Gradually Decreasing Their Precision. NeuroImage.

[45] Cattaneo Z., Vecchi T., Pascual-Leone A., Silvanto J. (2009). Contrasting Early Visual Cortical Activation States Causally Involved in Visual Imagery and Short-Term Memory. Eur. J. Neurosci..

